# The functional organization of the left STS: a large scale meta-analysis of PET and fMRI studies of healthy adults

**DOI:** 10.3389/fnins.2014.00289

**Published:** 2014-09-11

**Authors:** Einat Liebenthal, Rutvik H. Desai, Colin Humphries, Merav Sabri, Anjali Desai

**Affiliations:** ^1^Department of Neurology, Medical College of WisconsinMilwaukee, WI, USA; ^2^Department of Psychiatry, Brigham and Women's HospitalBoston, MA, USA; ^3^Department of Psychology, University of South CarolinaColumbia, SC, USA

**Keywords:** functional organization, superior temporal sulcus (STS), left hemisphere, meta-analysis, functional magnetic resonance imaging (fMRI), positron emission tomography (PET), speech perception, semantic processing

## Abstract

The superior temporal sulcus (STS) in the left hemisphere is functionally diverse, with sub-areas implicated in both linguistic and non-linguistic functions. However, the number and boundaries of distinct functional regions remain to be determined. Here, we present new evidence, from meta-analysis of a large number of positron emission tomography (PET) and functional magnetic resonance imaging (fMRI) studies, of different functional specificity in the left STS supporting a division of its middle to terminal extent into at least three functional areas. The middle portion of the left STS stem (*f*mSTS) is highly specialized for speech perception and the processing of language material. The posterior portion of the left STS stem (*f*pSTS) is highly versatile and involved in multiple functions supporting semantic memory and associative thinking. The *f*pSTS responds to both language and non-language stimuli but the sensitivity to non-language material is greater. The horizontal portion of the left STS stem and terminal ascending branches (*f*tSTS) display intermediate functional specificity, with the anterior-dorsal ascending branch (*f*atSTS) supporting executive functions and motor planning and showing greater sensitivity to language material, and the horizontal stem and posterior-ventral ascending branch (*f*ptSTS) supporting primarily semantic processing and displaying greater sensitivity to non-language material. We suggest that the high functional specificity of the left *f*mSTS for speech is an important means by which the human brain achieves exquisite affinity and efficiency for native speech perception. In contrast, the extreme multi-functionality of the left *f*pSTS reflects the role of this area as a cortical hub for semantic processing and the extraction of meaning from multiple sources of information. Finally, in the left *f*tSTS, further functional differentiation between the dorsal and ventral aspect is warranted.

## Introduction

The human superior temporal sulci occupy an important fraction of the temporal cortex, strategically located at the junction of major temporal—parietal and—frontal functional pathways. Portions of the superior temporal sulcus (STS) in each hemisphere have been assigned numerous specialized perceptual and cognitive functions (Hein and Knight, 2008). Given the size and orientation of the STS, a division along its anterior-to-posterior axis is predicted, but determination of the functional boundaries remains hotly debated. Anatomically, the STS in each hemisphere has been divided into a forward stem composed of an anterior, a middle, a posterior and an horizontal segment, and a backward ascending branch bifurcated into an anterior-dorsal and a posterior-ventral segment, based on 3D morphology and ontogenic observations (Ochiai et al., 2004). In the left hemisphere, structural and functional connectivity patterns to the inferior frontal cortex support a division of the superior temporal cortex into at least two, and perhaps three, segments that are part of functionally distinct anterior-ventral and posterior-dorsal streams for language processing (Frey et al., 2008; Saur et al., 2008; Rauschecker and Scott, 2009; Rauschecker, 2011; Turken and Dronkers, 2011), reminiscent of the dual stream model of auditory perception (Rauschecker and Tian, 2000). Functional neuroimaging data also suggests that the left STS can be divided along its anterior-to-posterior axis, with the left middle STS consistently associated with speech perception (Liebenthal et al., 2005; Obleser et al., 2007; Leaver and Rauschecker, 2010; DeWitt and Rauschecker, 2012) and more posterior areas associated with multiple functions including semantic processing (Dronkers et al., 2004), audiovisual integration (Calvert et al., 2001; Beauchamp, 2005), biological motion processing (Puce et al., 1998) and phonological processing (Wise et al., 2001; Buchsbaum et al., 2005; Liebenthal et al., 2010, 2013). However, the different functions associated with different portion of the left STS have seldom been localized and compared within the same set of subjects and experimental framework. Previous studies of the STS have compared pairs of similar functions within a cognitive domain, such as for example voice and speech recognition (Belin et al., 2000), speech perception and phonological processing (Liebenthal et al., 2010), or auditory, visual and somatosensory integration (Beauchamp et al., 2008). But, to our knowledge, systematic functional comparisons have not been carried out between multiple functions across cognitive domains (for example, between several language and non-language functions). As a result, the number and boundaries of distinct functional regions in the left STS remain to be determined.

Despite a remarkable growth in neuroimaging research in recent years, another persistent limitation to understanding the neuroanatomical organization of cognitive functions is that most studies rely on relatively small sample sizes and narrow experimental designs (i.e., a restricted number of experimental conditions). This is problematic because of the well-known inter-individual variability in brain structure, brain function, and brain structure-function relationships, including in the STS (Sowell et al., 2002; Kanai et al., 2012; Gilaie-Dotan et al., 2013). Particularly in the terminal aspect of the STS, the number of ascending branches and how they join the STS stem was found to be highly variable between individuals, causing irregularity in naming convention and contributing to the murkiness in functional characterization of this area (Segal and Petrides, 2012). Further challenging the characterization of terminal STS is the high degree of variability in the neighboring inferior parietal lobule (IPL), where the supramarginal gyrus (SMG) and angular gyrus (AG) were found to be composed of several distinct cytoarchitectural areas, suggestive of functional differentiation, with no consistent correspondence between cytoarchitectural and macroanatomical borders (Caspers et al., 2006). It is therefore valuable to examine brain activation patterns *across* neuroimaging studies in order to identify reliable functional organization principles in larger subject samples and in a wide array of cognitive paradigms.

Previous meta-analyses involving the temporal cortex have most often centered on one specific cognitive function, for example speech perception (Turkeltaub and Coslett, 2010), semantic processing (Binder et al., 2009; Adank, 2012), auditory attention (Alho et al., 2014), writing (Purcell et al., 2011; Planton et al., 2013), motion perception (Grosbras et al., 2012), emotion processing (Lee and Siegle, 2012), and theory of mind (Van Overwalle and Baetens, 2009). One prior meta-analysis focused on the multi functionality of the STS, but was limited to just a few studies per functional category that used similar stimuli and experimental designs (Hein and Knight, 2008).

**Figure 1:**
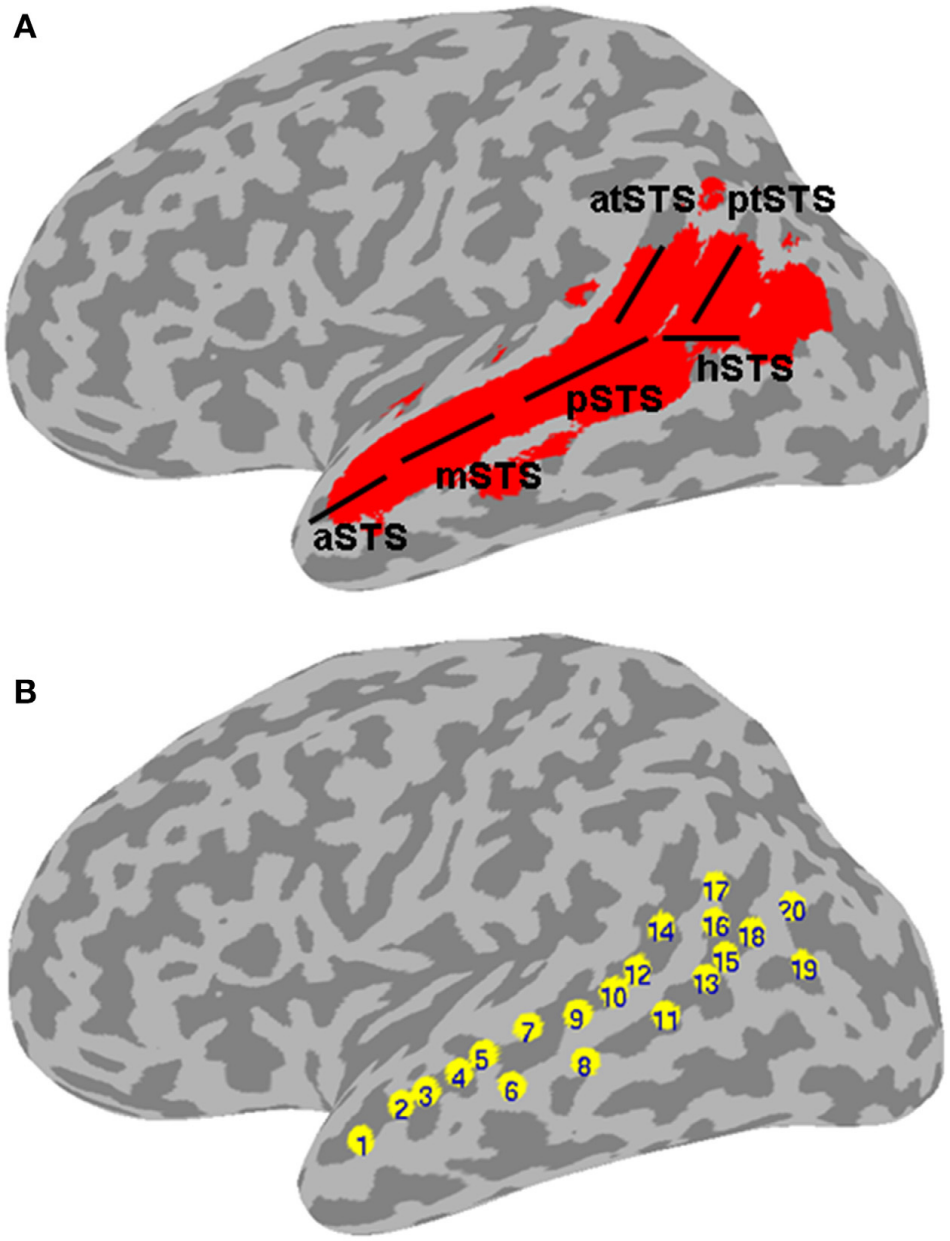
**Left STS probabilistic mask and ROIs. (A)** Probabilistic mask of the left STS (in red) shown projected onto a cortical surface model of the Colin brain in Talairach space. Also shown is a schematic approximation of the STS anatomical subdivisions used to describe the results (based on Ochiai et al., 2004), consisting of the aSTS, anterior STS; mSTS, middle STS; pSTS, posterior STS; hSTS, horizontal STS; atSTS, anterior branch of terminal STS; and ptSTS, posterior branch of terminal STS; **(B)** twenty evenly spaced spherical ROIs in the left STS, in which functional specificity was probed, shown projected onto the same cortical surface. The ROIs are numbered in ascending order according to their anterior-posterior position along the left STS.

The present meta-analysis was designed to study the functional organization of the left STS for language and non-language processing. The meta-analysis deliberately included a large number of studies using different neuroimaging methods (PET, fMRI), experimental designs (implicit, explicit, or no task), and stimuli (linguistic, nonlinguistic). The extent of the left STS was determined based on a probabilistic map created from structural magnetic resonance (MR) images of 61 brains. We reasoned that (1) drawing from commonalities in activation across multiple data sets generated using different experimental designs and methodologies would highlight reliable and fundamental functional organization patterns; and (2) defining the extent of the STS and a comprehensive set of putative STS functional categories would serve as a unifying platform for analyzing results from multiple studies, irrespective of anatomical labeling practices and interpretation of functional activation patterns across the studies. The reported results rely on analysis of 485 activation peaks from 253 studies that fell within the left STS mask. The peaks were sorted into 2 stimulus categories and 15 functional categories based on the experimental contrast used to generate each activation map. The main results are reported in terms of functional specificity, expressed as the number of stimulus and functional categories with a significant mean activation likelihood estimate, in different areas of the left STS. Structural subdivisions of the STS are labeled using an approximation of the demarcation of Ochiai et al. (2004), as detailed schematically in Figure 1A. Note that the anterior-dorsal ascending branch of the terminal STS (atSTS) is immediately posterior to the ascending branch of the Sylvian fissure. The atSTS is expected in most brains to be anterior to the first intermediate sulcus of Jensen, which (when present) is considered to form the boundary between the SMG and AG (Caspers et al., 2006; Segal and Petrides, 2012). As such, the atSTS terminates in most brains within the posterior SMG, near the boundary with AG. The posterior-ventral branch of the terminal STS (ptSTS) terminates within the AG.

## Materials and methods

A probabilistic map of the left STS was created by averaging two T1-weighted MR images from each of 61 brains, in which the STS had been demarcated using Freesurfer (Dale et al., 1999) for automatic parcellation of sulci and gyri (Destrieux et al., 2010). The resulting STS atlas (labeled TT_desai_ddpmaps) is included with AFNI (Cox, 1996). The left STS probabilistic map was thresholded at 20% probability and extended 5 mm laterally to create a mask for the meta-analysis (Figure 1A). Note that the STS, as parcellated in the Destrieux et al. atlas, broadens toward the posterior end and arguably includes parts of the posterior middle temporal gyrus (pMTG), AG, and possibly SMG. We chose to use the same parcellation for consistency and to ensure adequate sampling of activation in the terminal STS.

In the BrainMap database (Laird et al., 2005), 675 PET and fMRI studies published in the years 1990–2010 were identified that reported activation peaks located within the left STS mask, as assessed based on reported coordinates in Talairach space (Talairach and Tournoux, 1988). From these, 485 activation peaks from 253 different studies meeting the inclusion criteria of representing data collected from a group of at least 8 healthy adults of mixed gender, and using a high-level baseline, were incorporated in the meta-analysis. Functional contrasts using a low-level baseline, such as fixation or rest, were excluded due to the uncertainty associated with the nature of activations in such comparisons.

Each activation peak was categorized according to the type of stimulus material and the functional contrast used to generate the activation. The stimulus categories consisted of “language” (including auditory and visual spoken, or written, sub-syllabic, syllabic, word, sentence or discourse stimuli) and “non-language” (including all types of non-verbal and non-written stimuli not included in the language category). The functional categories consisted of 15 sensory, motor, or cognitive processes most commonly targeted by the condition contrasts used to generate the peaks included in the meta-analysis. The functional categories were further classified as linguistic or non-linguistic for the purpose of comparing each functional category with the other categories in its class. The complete list of stimulus and functional categories, and functional classes, is given in Table 1.

**Table 1:**
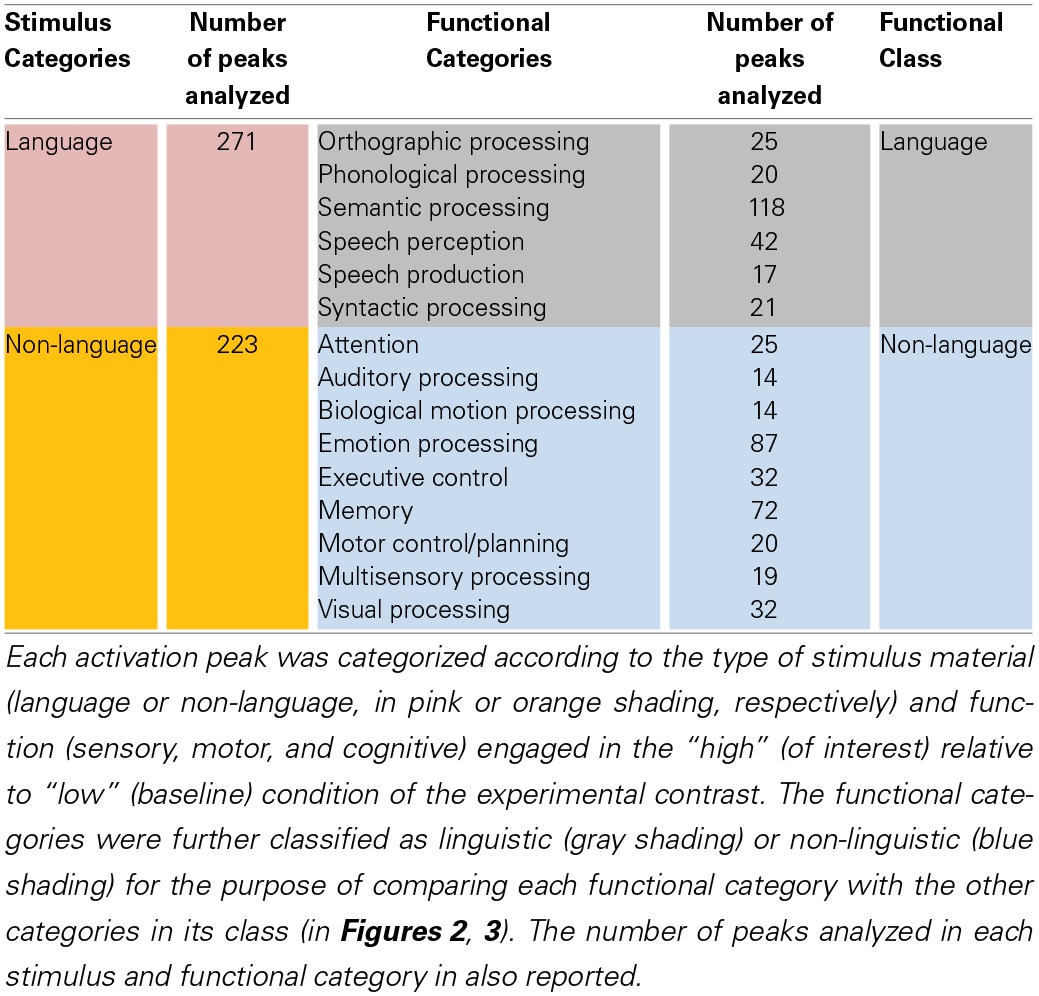
**Stimulus and functional categories used to sort the left STS activation peaks**.

**Figure 2:**
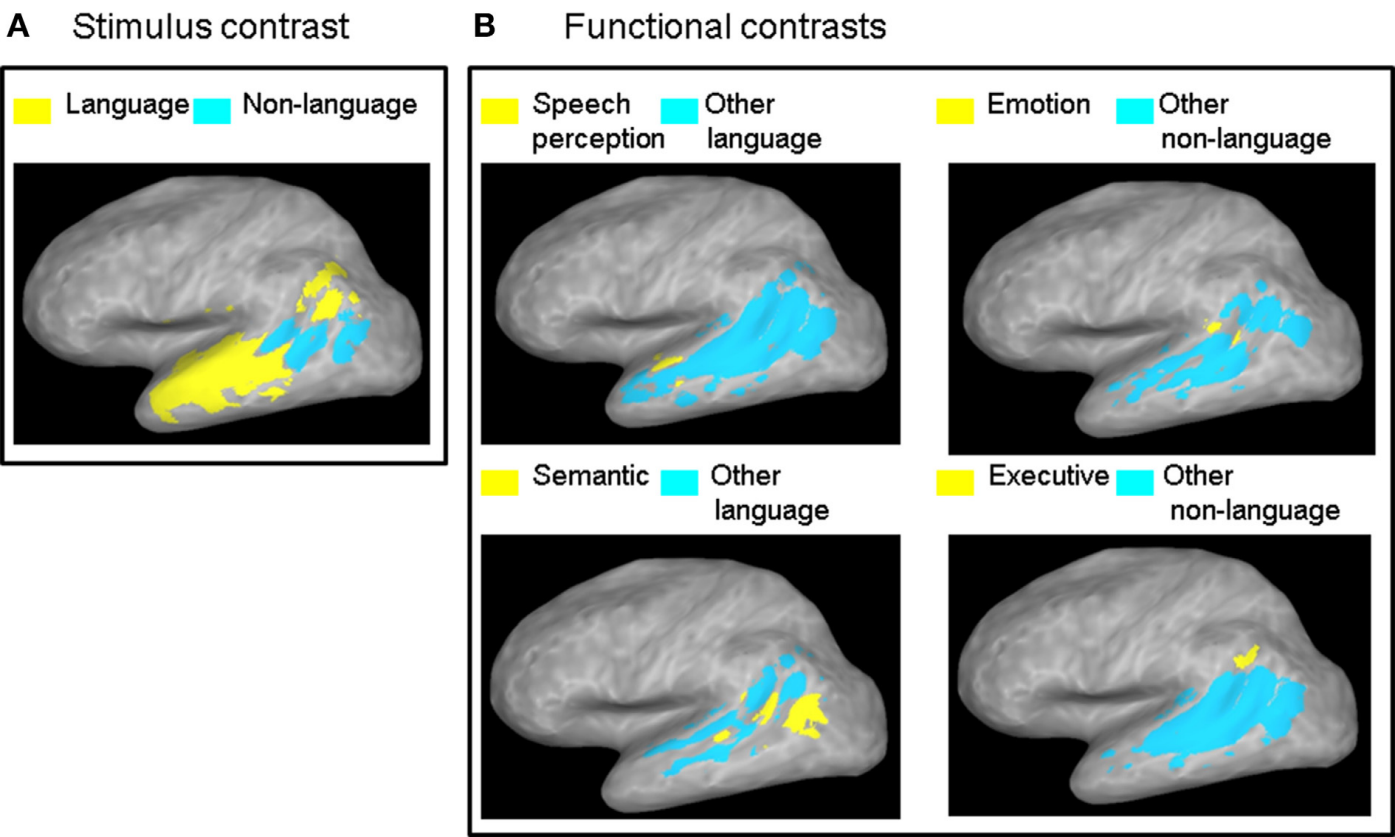
**ALE contrast maps.** Contrast maps of **(A)** the two stimulus categories and **(B)** each functional category relative to all the other functional categories in the same class. Maps are thresholded at a corrected probability of α < 0.05. Only functional contrasts resulting in significant differential ALE measurement in the left STS are displayed.

**Figure 3:**
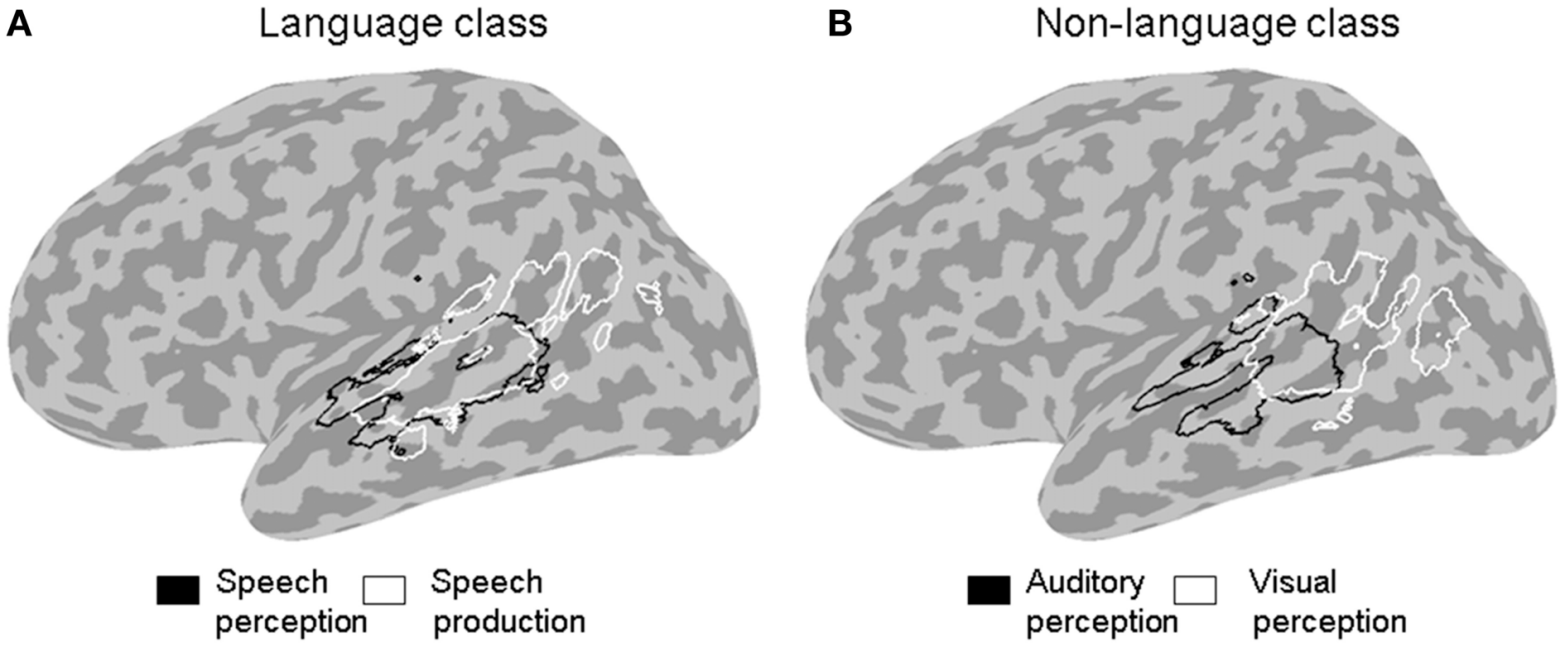
**ALE outline maps.** Outline maps of pairs of functional categories in the language **(A)** and the non-language **(B)** functional classes that showed significant differences in activation likelihood in the left STS at a corrected error probability of α < 0.05.

Each activation peak was categorized according to the type of stimulus material (language or non-language, in pink or orange shading, respectively) and function (sensory, motor, and cognitive) engaged in the “high” (of interest) relative to “low” (baseline) condition of the experimental contrast. The functional categories were further classified as linguistic (gray shading) or non-linguistic (blue shading) for the purpose of comparing each functional category with the other categories in its class (in Figures 2, 3). The number of peaks analyzed in each stimulus and functional category in also reported.

Peaks were assigned to a stimulus category based on the input material used in the “high” (of interest) compared to “low” (baseline) condition of the experimental contrast, and to up to three different functional categories representing the main sensory, motor, or cognitive functions considered to be engaged in the “high” relative to “low” condition of the contrast. For example, an activation peak resulting from a perceptual contrast of clear spoken sentences and non-intelligible speech-like sounds would be assigned to the language stimulus category and to the functional categories of speech perception, semantic processing, and syntactic processing. There were 271 and 223 peaks assigned to the language and non-language stimulus categories, respectively. Sixteen peaks were assigned to both the Language and Non-Language stimulus categories. These peaks resulted from contrasts in which the stimuli used in the “high” condition contained both linguistic and non-linguistic information that was not balanced by the stimuli used in the “low” condition. For example, some studies of audiovisual speech perception compared a video clip of a face producing natural speech with a series of stilled frames of the face showing apical gestures (Calvert and Campbell, 2003). The differential activation in this contrast was considered to reflect the higher linguistic (speech) and non-linguistic (biological motion) content of the stimuli in the “high” condition. Seven peaks were not assigned to either Language or Non-language stimulus categories. These peaks resulted from contrasts in which no stimulus was used in the “high” condition. For example, some studies compared an internal task such as imagination, in which no external stimulus was used, with a perceptual task (Kosslyn et al., 1996). Such peaks were assigned to functional categories and were included in comparisons between functional (but not stimulus) categories. The number of peaks assigned to each functional category (reported in Table 1) ranged 14–118 (mean = 37), with “semantic processing” as the largest category. The degree of overlap in peak assignment between pairs of functional categories ranged 0.03–0.52 (mean = 0.23), with the largest overlap occurring between “orthographic processing” and “semantic processing.”

The GingerALE version 2.0.4 application of the BrainMap software was used to perform the meta-analysis, with fixed 10 mm FWHM Gaussian smoothing (Turkeltaub et al., 2002; Eickhoff et al., 2009, 2012). The activation likelihood estimation (ALE) technique estimates the convergence of neuroimaging activation foci by modeling them as Gaussian probability distributions based on assessment of spatial uncertainty due to intersubject and co-registration variability. A relatively low and fixed (i.e., not adjusted according to study sample size) level of smoothing was used in order to maintain sensitivity to potential small subdivisions within the STS and to avoid potential bias from systematic differences in study sample sizes across functional categories. The ALE in the two stimulus categories was compared (Figure 2A). The ALE in each functional category was compared with the ALE in *all* other functional categories in the same class (Figure 2B), and also with the ALE in *each* of the other functional categories in the same class in a pairwise fashion (Figure 3), where class was defined as language or non-language (see Table 1). The ALE category contrast maps for the entire left STS were thresholded at *p* < 0.01 and clusters smaller than 700 μl were removed, resulting in a corrected error probability of α < 0.05, as determined using the AlphaSim module in AFNI (Ward, 2000).

**Figure 4:**
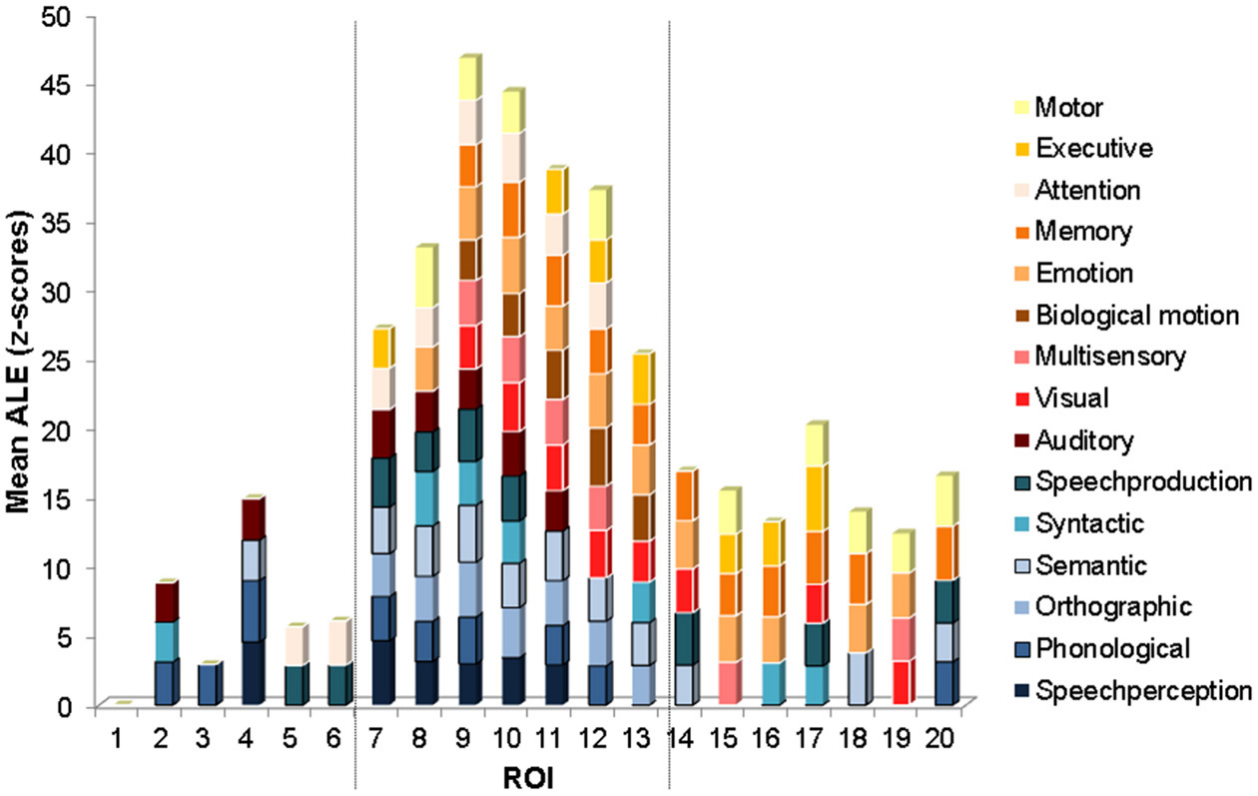
**ROI mean ALE functional measure.** Graph of mean ALE measure within each ROI for each functional category exceeding the significance level (mean *z* = 2.807, *p* < 0.005). The categories are stacked according to functional class, with the language functional categories on the bottom in shades of blue, and the non-language functional categories on the top in shades of red and orange. The ROIs are ordered from the most anterior (ROI 1) to the most posterior (ROI 20) along the STS (see Figure 1B for the anatomical location of each ROI). The vertical dashed lines show locations of marked changes in functional specificity.

**Figure 5:**
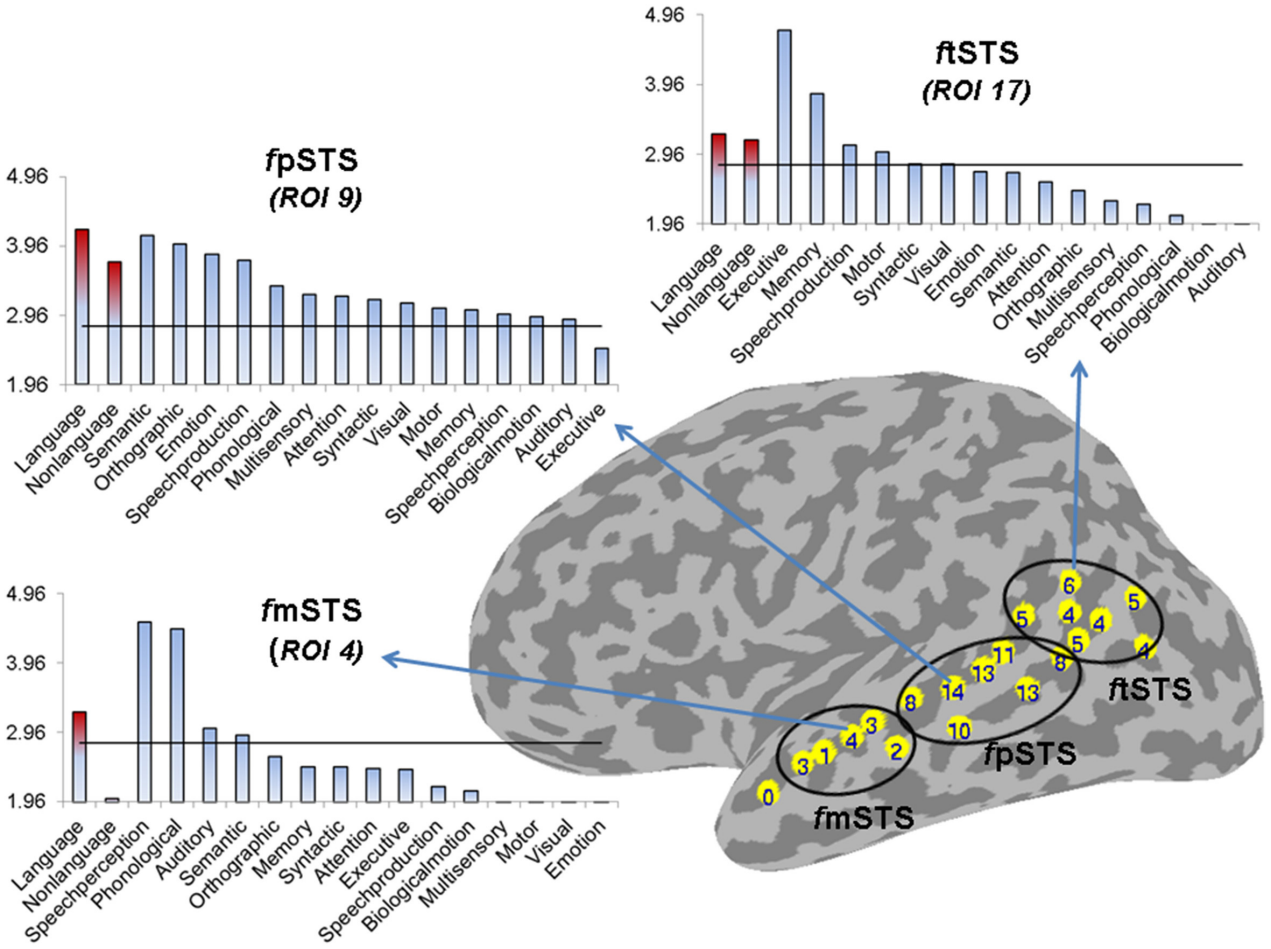
**Partition of left STS into three subdivisions based on functional specificity.** The number label within each ROI represents its functional specificity, expressed as the number of functional categories with a significant mean ALE measure in this region (*p* < 0.005). The functional mSTS (*f*mSTS) subdivision was defined as a region activated by a small number of functional categories (range 1–4, mean 2.6), the functional pSTS (*f*pSTS) subdivision was defined as a region activated by the largest number of functional categories (range 8–14, mean 11), and the functional tSTS (*f*tSTS) subdivision was defined as a region activated by an intermediate number of functional categories (range 4–6, mean 4.7). The three graphs show the mean ALE measure (expressed in Z-scores) for each stimulus (in red) and functional (in blue) category in descending order of magnitude, in the ROIs that were activated by the largest number of functional categories in each subdivision (ROIs number 4, 9, and 17 in the left *f*mSTS, *f*pSTS, and *f*tSTS, respectively). The horizontal line corresponds to *z* = 2.807 (*p* < 0.005).

In a second analysis, the functional organization of the left STS was studied in finer grain by using a region of interest (ROI) approach. The left STS mask was divided into twenty ROIs. Because the geometry of the STS does not follow a straight line, we used a clustering algorithm to partition the left STS mask into twenty sub-regions that were approximately equal-sized and evenly spaced. This was accomplished by submitting the x, y, z coordinates of all the voxels in the mask to a k-means clustering algorithm set to identify twenty clusters. The cluster center coordinates were then used as the center positions of twenty 4 mm-radius spherical ROIs. The location of ROIs within the left STS mask is shown in Figure 1B. The mean ALE (expressed in *z*-scores) within each ROI was calculated for each functional category. The functional specificity of each ROI was estimated by tallying the number of categories activating this region at *p* < 0.005 (*z* > 2.807). Results of the ROI analyses are shown in Figures 4, 5.

The cortical inflated surfaces in Figure 2 were rendered using Caret 5.62 (Van Essen et al., 2001). The surfaces in the other figures were rendered using custom code in Matlab (Matlab 7.1, The Math Works Inc., Natick, MA).

## Results

The contrast between the two stimulus categories (Figure 2A) showed a greater likelihood of language compared to non-language activation peaks in most of the left STS, except in the posterior and horizontal STS stem (pSTS and hSTS, respectively) where a greater likelihood of non-language activation peaks was observed. The contrasts between each functional category in the language class and all the other categories in that class (Figure 2B, left panels) revealed significantly greater likelihood of speech perception peaks in the middle STS stem (mSTS), and of semantic processing peaks in pSTS and hSTS. The contrasts between each functional category in the non-language class and all the other categories in that class (Figure 2B, right panels) revealed significantly greater likelihood of emotion processing peaks in pSTS, and of executive processing peaks in the anterior terminal STS branch (atSTS). The non-language area in the stimulus contrast (Figure 2A) overlapped considerably with the semantic and emotion areas in the functional contrasts (Figure 2B). Pairwise comparisons between the functional categories in each class (Figure 3) revealed greater likelihood of speech perception peaks in mSTS relative to greater likelihood of speech production peaks in the anterior (atSTS) and posterior (ptSTS) terminal STS branches, as well as greater likelihood of auditory perception peaks in mSTS relative to greater likelihood of visual perception peaks in pSTS and hSTS. Other functional contrasts resulted in no significant differences (α < 0.05).

The ROI analysis revealed the functional properties of the left STS with greater spatial detail. The mean ALE within each ROI, for each of the functional categories is plotted in Figure 4. Several interesting observations arise from this analysis. The most anterior ROIs (numbered 2–6) show significant activation likelihood for just a few functional categories (range 1–4, mean 2.6) largely from the language class (in shades of blue). The ROIs in intermediate position (numbered 7–13) show significant activation likelihood for the largest number of functional categories (range 8–14, mean 11) from both the language and non-language classes (the latter in shades of red and orange). The ROIs in the most posterior part of the left STS (numbered 14–20) show significant activation likelihood for an intermediate number of functional categories (range 4–6, mean 4.7) from both the language and non-language classes. The difference in functional specificity (expressed as the number of functional categories with a significant mean ALE measure) between the three regions is significant [One-Way ANOVA, *F*_(2, 16)_ = 43, *p* = 0]. Anatomically, the anterior ROIs (2–6) correspond roughly to the mSTS stem area, the intermediate ROIs (7–13) correspond roughly to the pSTS stem area, and the most posterior ROIs correspond roughly to the hSTS stem area and the atSTS and ptSTS branches. Note that in ROI 1, none of the categories survived the statistical threshold, likely due to a small number of activation peaks falling within this area.

Based on these differences in functional specificity, we propose a division of the left STS into middle, posterior and terminal functional areas, labeled respectively *f*mSTS (talairach y coordinates −7 to −27), *f*pSTS (talairach y coordinates −28 to −59), and *f*tSTS (talairach y coordinates −55 to −71). Figure 5 shows an approximate demarcation of the three functional areas and their specificity, as well as plots of the mean ALE measure for each stimulus and functional category in the ROIs activated by the largest number of functional categories (i.e., the least specific ROIs) in each sub-division. In the *f*mSTS, the least functionally specific ROI (number 4) showed significant activation likelihood only for language stimuli, and only for the speech perception, and phonological, auditory, and semantic processing functional categories. In the *f*pSTS, the least specific ROI (number 9) showed significant activation likelihood for both language and non-language stimuli, and for 14 out of the 15 possible functional categories (with the exception of executive control). In the *f*tSTS, the least functionally specific ROI (number 17) showed significant activation likelihood for both language and non-language stimuli, and for the executive and motor control, memory, speech production, and syntactic and visual processing functional categories.

With regard to *f*tSTS, despite the similar level of functional specificity across this area, we expect that it is composed of an anterior and a posterior subdivision (*f*atSTS and *f*ptSTS, respectively), based on its irregular 3D anatomy and apparent dichotomous functionality related primarily to executive control in atSTS and to semantic processing in hSTS and ptSTS (see Figure 2).

Several potential limitations should be mentioned with respect to the results. First, the Brainmap database is not a random sample of the neuroimaging literature and may be biased toward studies of certain cognitive functions. For example, the smaller number of studies of speech perception (42) compared to studies of semantic processing (118) found here with peaks falling in the left STS may reflect a sampling bias in the database or a true aspect of STS functional organization. Seconds, the distribution of number of peaks analyzed was not even along the left STS, with fewer peaks falling in the mSTS area (66) and more peaks falling in the pSTS (224) and tSTS (195) areas. Importantly, the difference in the distribution of the number of peaks along the STS cannot in itself explain the higher functional specificity of the mSTS because the distribution of the number of peaks was not random with respect to functional and stimulus category. That is, not all the stimulus and functional categories were evenly less represented in mSTS relative to pSTS and tSTS. On the contrary, a small number of categories were actually better represented in mSTS than in the rest of the STS. In particular, the category of speech perception had higher ALE values than all of the other language categories combined specifically in mSTS (Figure 2B), and the mSTS showed higher ALE values for Language over Non-Language stimuli (Figure 2A).

## Discussion

We present here new evidence from meta-analysis of a large number of PET and fMRI studies, of different functional specificity along the left STS supporting a division of its middle to terminal extent into at least three functionally distinct areas. Based on the present results, and a review of the literature, we suggest that a functional area in the left middle STS (*f*mSTS; Talairach y coordinates −7 to −27) is highly specialized for speech perception and the processing of language material. A functional area in the left posterior STS (*f*pSTS; Talairach y coordinates −28 to −59) is highly versatile and serves as a hub for semantic processing and multiple functions supporting semantic memory and associative thinking. The *f*pSTS responds to both language and non-language stimuli but the likelihood of response to non-language material is greater. A functional area including the left horizontal and terminal STS (*f*tSTS; Talairach y coordinates −55 to −71) displays intermediate functional specificity, with the anterior ascending branch adjoining SMG (*f*atSTS) supporting executive functions and motor planning and showing greater likelihood of response to language material, and the horizontal stem and posterior ascending branch adjoining AG (*f*ptSTS) supporting primarily semantic processing and displaying greater likelihood of response to non-language material. These latter results in the *f*tSTS suggest that a further functional differentiation between its dorsal and ventral aspect is warranted.

The finding of a strong convergence of activity related to speech processing in the left *f*mSTS is largely consistent with prior neural functional models associating this area with phonemic perception (Davis and Johnsrude, 2003; Liebenthal et al., 2005; Obleser et al., 2007; Leaver and Rauschecker, 2010; DeWitt and Rauschecker, 2012). The left mSTS is considered to be part of a ventral auditory pathway for speech recognition, connecting the auditory cortex to semantic regions widely distributed in the left middle and inferior temporal cortex. Neurons in the left mSTS may be specially tuned to the categorical properties of native speech phonemes (Liebenthal et al., 2005; Leaver and Rauschecker, 2010; Humphries et al., 2013) making this area critical for decoding incoming speech signals. The most novel and striking aspect of the current results is the narrow functional specificity of the left *f*mSTS, observed as significant preference to language over non-language stimuli and to speech perception over other language functions (Figures 2, 3), as well as the convergence of peaks from only a few functional categories mostly in the language class (Figures 4, 5), in this area. It is possible that the high functional specificity of the left *f*mSTS for speech is an important means by which the human brain achieves its exquisite affinity and efficiency for native speech perception. The anatomical proximity of the mSTS to auditory cortex, and higher sensitivity of this region to auditory over visual processing (Figure 3), are also consistent with a specialization in this area for speech perception over other (non-auditory based) language functions.

The finding of a strong convergence of activity related to semantic processing in the left *f*pSTS is consistent with prior work indicating the importance of the adjacent left posterior MTG (pMTG) to language comprehension (Price, 2000, 2010; Dronkers et al., 2004; Binder et al., 2009). Lesions in the left pMTG are known to be particularly detrimental to language comprehension (Boatman et al., 2000; Dronkers et al., 2004; Baldo et al., 2013). The left posterior superior temporal cortex is activated during language comprehension irrespective of the input modality, including during sign language processing in native signers (Bavelier et al., 1998; MacSweeney et al., 2006). The main novel aspect of the present results is again related to functional specificity, which was astonishingly low in the left *f*pSTS and in sharp contrast to the high functional specificity observed in the left *f*mSTS. The left *f*pSTS was found to be extremely multi-functional, being more likely to respond to non-language stimuli, during semantic and emotion processing over other language and non-language functions, respectively (Figure 2); but also likely to respond to language stimuli and to almost all other functional categories (Figures 4, 5). The observation that an area “specializing” in semantic processing is overall more responsive to non-linguistic (i.e., non-verbal and non-written) stimuli is perhaps not intuitive. However, this finding is consistent with the idea that the very nature of semantic processing involves association of input from the different senses, analyzed in various ways (e.g., sensory features, biological motion, emotional valence, etc…), to extract information relevant to object recognition and comprehension. The extreme multi-functionality of the left *f*pSTS may reflect the role of this area as a cortical hub for semantic processing and the extraction of meaning from multiple sources of information. The strategic location of the left *f*pSTS, at the confluence of auditory and visual afferent streams, and fronto-parietal somato-motor and executive control efferent streams, is ideal for a cortical hub, in line with the concept of a neural convergence zone (Damasio, 1989; Meyer and Damasio, 2009) or epicenter (Mesulam, 1990, 1998).

The finding of a mixed pattern of functionality in the left *f*tSTS is perhaps not surprising given the complex anatomy of this area and varied functionality of bordering areas. The atSTS branch terminates near the SMG, an area suggested to serve as an auditory-motor interface (Guenther et al., 2006; Hickok and Poeppel, 2007), whereas the ptSTS branch terminates into the AG, an area associated primarily with semantic processing (Binder et al., 2009; Price, 2010). The preference observed here of the left *f*atSTS for language stimuli and executive and motor control functions (Figures 2, 3) is well in line with the implication of this and the neighboring SMG area in phonological processing (Paulesu et al., 1993; Caplan et al., 1997; Wise et al., 2001; Buchsbaum et al., 2005; Buchsbaum and D'Esposito, 2009; Liebenthal et al., 2013) and the learning of ambiguous or non-native sound categories (Callan et al., 2004; Golestani and Zatorre, 2004; Raizada and Poldrack, 2007; Desai et al., 2008; Liebenthal et al., 2010; Kilian-Hutten et al., 2011). The *f*atSTS may be important for maintenance of auditory sequences in short-term memory while their auditory, somatosensory, and motor properties are analyzed to support phonemic perception. In contrast, the preference observed here of the left *f*ptSTS for non-language stimuli and semantic processing bears resemblance to the preference of the nearby *f*pSTS area, and is well in line with the implication of the AG in semantic retrieval and semantic integration (Price, 2000, 2010; Dronkers et al., 2004; Binder et al., 2009; Binder and Desai, 2011; Bonner et al., 2013). The left *f*ptSTS area could be an extension of the left *f*pSTS semantic area identified here and a functional bridge to the AG. Taken together, these results support a functional differentiation between the anterior-dorsal and posterior-ventral aspects of tSTS, in line with the different role of dorsal and ventral portions of the IPL. Nevertheless, given the documented high intersubject variability in terminal STS, caution should be used in treating differences in activation within this area and with the adjacent IPL. The functional differentiation within terminal STS should be addressed further in future work, perhaps taking into account cyoarchitectural information.

Structural connectivity and resting state functional connectivity patterns in the left temporal cortex are also in line with a left STS anterior-to-posterior segregation based on functional specificity. Disparate language pathways are thought to connect the left middle and posterior superior temporal cortex with the inferior frontal gyrus (IFG), consistent with ventral and dorsal streams of processing for language (Saur et al., 2008; Rauschecker and Scott, 2009; Rauschecker, 2011). Structural connectivity measured with diffusion tensor imaging showed that the middle superior temporal cortex is connected to the anterior IFG via the ventral portion of the extreme capsule fiber system and also via the uncinate fasciculus. In contrast, the posterior superior temporal cortex is connected to the posterior IFG directly via the arcuate fasciculus, and also indirectly through the inferior parietal cortex via the superior longitudinal fasciculus (Catani et al., 2005; Parker et al., 2005; Anwander et al., 2007; Frey et al., 2008). The left pMTG was found to have particularly rich structural connections with other brains areas through several major pathways connecting it to the AG and to the rest of the temporal cortex, in addition to IFG (Turken and Dronkers, 2011). Similarly, resting state functional connectivity in the left middle superior temporal cortex was found to be limited to the posterior temporal cortex and the IFG (Turken and Dronkers, 2011). In contrast, functional connectivity in the left pMTG was found to be among the highest in the cerebral cortex, with connections to the left AG, anterior STG, and IFG (Buckner et al., 2009; Turken and Dronkers, 2011). The locus of most extensive functional connectivity in the left pMTG indicated in the Buckner study (Talairach x, y, z coordinates −62, −38, −12) coincides with the anterior-posterior position of the pSTS area of least functional specificity observed in the present study (ROI 9, Talairach x, y, z coordinates −48, −39, −1).

The current STS meta-analysis extends that of Hein and Knight (2008) by introducing a new functional specificity measure highlighting the organization of the left STS for language and non-language processing. This new perspective was possible mainly thanks to the much larger number of studies across language and non-language domains analyzed here. In the Hein and Knight study, activation peaks in the speech perception category were clustered in the anterior portion of the STS (approximately corresponding to the mSTS area described here), whereas those for several other categories (multisensory processing, biological motion processing) were clustered in the posterior portion the STS (approximately corresponding to the pSTS and tSTS areas described here) though with a small presence also in the anterior STS. The results were interpreted as different degrees of multi-functionality in the anterior and posterior STS rather than a functional differentiation *per se*, because there was some degree of spatial overlap between functional categories along the entire STS. The present meta-analysis supports the concept of differences in multi-functionality along the STS. But the extreme low multi-functionality in the mSTS and contrastingly extreme high multi-functionality in the adjacent pSTS observed here suggest that there may be fundamental differences between these areas reflecting a true functional specialization for speech perception and semantic processing, respectively, rather than merely a gradient of multi-functionality.

In conclusion, the present work demonstrated a division of the mid-to-terminal left STS into at least three functional areas based on functional specificity. Future work using a more detailed definition of stimulus and functional categories, as well as finer anatomic parcellation of the STS mask, may yield further insights into the functional organization of left STS and the interaction of each functional subdivision with neighboring regions. A comparison with the functional organization of the right STS is also warranted.

### Conflict of interest statement

The authors declare that the research was conducted in the absence of any commercial or financial relationships that could be construed as a potential conflict of interest.
